# 肺癌切除术后肺内孤立性转移复发灶的射频消融治疗

**DOI:** 10.3779/j.issn.1009-3419.2014.06.04

**Published:** 2014-06-20

**Authors:** 宝东 刘, 磊 刘, 牧 胡, 坤 钱, 元博 李, 修益 支

**Affiliations:** 100053 北京，首都医科大学宣武医院胸外科 Department of Thoracic Surgery, Xuanwu Hospital, Capital Medical University, Beijing 100053, China

**Keywords:** 肺肿瘤, 转移, 复发, 射频消融, Lung neoplasms, Metastases, Recurrence, Radiofrequency ablation

## Abstract

**背景与目的:**

肺癌是最常见的恶性肿瘤之一，外科手术仍是早期非小细胞肺癌的首选治疗手段，然而术后复发降低了患者的生存预期。但是部分患者如局部复发或孤立性肺内转移通过局部治疗可以延长生存甚至治愈。射频消融术（radio-frequency ablation, RFA）成为一种不适合手术治疗的原发性或转移性肺肿瘤的新的局部治疗手段。本研究的目的是评价非小细胞肺癌切除术后肺内孤立性复发转移灶的治疗效果。

**方法:**

2008年12月-2013年11月对20例肺癌切除术后不能再次手术的孤立性肺内转移复发灶进行CT引导下射频消融术。男性15例，女性5例，年龄45岁-85岁，平均69.2±11.6岁。全组病例均有病理学证实（腺癌14例、鳞癌6例）。病灶直径最小2.0 cm，最大8.0 cm，平均3.9±2.0 cm。对其并发症、无进展生存（progression-free survival, PFS）和总生存期进行回顾性分析。

**结果:**

全组病例均能完成射频消融术，平均消融时间为34.3 min（15 min-60 min），术中常见的并发症是胸痛8例（40%），无围术期死亡。中位PFS为25.0个月；中位生存时间27.0个月，1年生存率为92.9%，2年生存率为57%。

**结论:**

RFA对非小细胞肺癌术后不能耐受再次手术的肺内孤立性转移复发安全可行。

## Abstract (en)

**Background and objective:**

Primary lung cancer is one of the most common malignancies worldwide. Surgical resection remains the first choice for the treatment of early stage non-small cell lung cancer (NSCLC). Relapse after surgery sharply reduces the patient's life expectancy. This relapse is referred to as isolated postsurgical local recurrences or metastases (IPSLROM), which can be treated via local therapy to achieve long-term survival or cure. In recent years, radiofrequency ablation (RFA) has been increasingly used as a non-surgical treatment option for patients with primary and metastatic lung tumors. This study aims to evaluate the efficacy of RFA among patients with IPSLROM of NSCLC.

**Methods:**

A total of 20 patients underwent computerd tomograghy (CT)-guided RFA for lung neoplasm with IPSLROM of NSCLC (with unresectable disease because of poor lung reserve or multifocality) in our hospital between December 2008 and November 2013. These patients comprised 15 males and 5 females with a mean age of 69.2 years (range: 45-85). All patients exhibited pathological evidence of neoplastic lesion (14 tumors were adenocarcinoma, and six were squamous cell carcinoma). The mean size of the lesions was 3.9 cm (range: 2.0 cm to 8.0 cm). Treatment complications, progression-free survival (PFS), and survival parameters were retrospectively analyzed.

**Results:**

RFA was well tolerated by all patients with an average time of 34.3 min (range: 15 min to 60 min). Intraprocedural complications included eight cases of chest pain (40%). No procedure-related deaths occurred in all of the 20 ablation procedures. The median PFS was 25 months in all of the patients who received RFA. The median overall survival for the entire group of patients was 27.0 months. No differences were observed in the overall survival between patients with IPSLROM. The overall survival rates at 1 and 2 years after RFA were 92.9% and 57.0%, respectively.

**Conclusion:**

RFA is a safe and effective procedure in unresectable lung tumors with IPSLROM of NSCLC.

肺癌是最常见的恶性肿瘤之一，外科手术仍是早期非小细胞肺癌（non-small cell lung cancer, NSCLC）患者（Ⅰ期、Ⅱ期和部分Ⅲa期）的首选治疗手段，但是大约30%的患者术后会出现局部或区域复发，是治疗失败的主要原因之一^[1-3]^。出现局部复发后再次手术治疗的难度较大，放射治疗成为这部分患者的主要治疗手段，但是由于受手术及低肺功能的影响，NSCLC患者术后对放射治疗的耐受性较差。近年来，射频消融术（radio-frequency ablation, RFA）成为一种不适合手术治疗的原发性或转移性肺肿瘤的新的局部治疗手段，但是对NSCLC切除术后肺内孤立性复发转移灶（isolated postsurgical local recurrences or metastases，IPSLROM，定义为肺内孤立性转移或复发，肺外无病灶）的研究还比较少，本文回顾性评价了RFA治疗IPSLROM的临床效果。

## 1 材料与方法

### 1.1 临床资料

2008年12月-2013年11月间首都医科大学宣武医院对肺癌切除术后不能再次手术或放疗的IPSLROM进行CT引导下RFA。

入组标准为：①影像学检查诊断为肺内孤立性转移或复发；②肺外无病灶；③治疗前获得病理学诊断（如CT引导下经皮肺穿刺活检），要求二次病理结果与首次一致；④不能耐受或拒绝手术或放疗，接受射频消融术。排除标准为：①合并远处转移；②复发转移距手术时间 < 6个月。

入组患者20例，其中男性15例，女性5例，年龄45岁-85岁，平均69.2±11.6岁。复发时体力状况评分采用ECOG（Eastern Cooperative Oncology Group）标准均在0分-2分。既往手术中肺叶切除术12例，因身体或年龄等因素选择肺楔形切除术8例。切除术后局部复发或转移时间11个月-132个月，平均63.3±46.4个月。局部复发8例，转移12例，其中同侧肺内转移6例，对侧肺内转移6例。复发或转移灶位于右上肺5例、右中肺2例、右下肺5例、左上肺4例、左下肺4例。病灶直径最小2.0 cm，最大8.0 cm，平均3.9±2.0 cm。病理学类型：腺癌14例、鳞癌6例。

### 1.2 射频消融术

局麻CT引导下，按照“四步法”穿刺定位^[4]^，使用250 W射频发生器和射频针（RITA产品：StarBurst XL，StarBurst Talon），靶温度设定为90 ℃，消融时间15 min-60 min，平均消融时间34.3 min。

再次CT扫描，观察病灶有无变化和气胸出血等并发症，确定患者无异常时返回病房，静卧2 h；预防性抗菌素使用，发热、咳血等给予对症处理。

### 1.3 综合治疗

根据病理标本作表皮生长因子受体（epidermal growth factor receptor, *EGFR*）基因检测，11例患者有突变推荐EGFR酪氨酸激酶抑制剂（tyrosine kinase inhibitor, TKI）治疗；另9例无突变推荐化疗，化疗采用铂类为基础的联合方案，包括紫杉醇、多西他赛、长春瑞滨、吉西他滨等，中位化疗周期数为4个（2个-5个）。

### 1.4 并发症评估

参照美国介入放射学会（Society Of Interventional Radiology, SIR）的分级标准^[5]^：①死亡：需要说明原因，与消融之间的关系；②严重并发症：导致死亡或者致残，需要住院或者临床处理，增加住院时间，包括输血、胸腔闭式引流。③轻微并发症：其他并发症，包括气胸、肿瘤种植；④副作用：指伴随治疗出现的不良结果，一般经常发生，但很少造成实际的损害，包括疼痛、胸膜反应、肺内出血、血痰、胸腔积液、消融后综合征。

### 1.5 随访

RFA术后的推荐每3-6个月随访1次。无病生存时间（progression-free survival, PFS）从治疗当天开始计算，终止日期为死亡时间或疾病进展日。生存期从治疗当天开始计算，终止日期为死亡时间或末次随访日。

### 1.6 统计学处理

采用SPSS 16.0统计软件进行数据统计分析，生存率采用*Kaplan-Meier*法计算，采用*Log-rank*检验比较生存差异。*P* < 0.05为差异有统计学意义。

## 2 结果

### 2.1 并发症

RFA无围手术期死亡，无相关严重并发症发生，术中一般感觉局部发热、出汗、甚至心率加快，无需特殊处理；8例患者术中疼痛（40%），经过镇痛等对症治疗好转，或者降低靶温度到70 ℃，经过数分钟后大部分患者靶温度可以逐渐升至90 ℃。术后大部分患者会出现消融后综合征，对症治疗后好转。

### 2.2 随访

所有患者随访截止2013年12月31日，平均随访时间为19个月（1个月-36个月），死亡9例，存活11例。全组中位PFS为25.0个月（图 1）。中位生存时间为27.0个月，1年生存率为92.9%，2年生存率为57%（图 2）。肺内孤立性转移组（12例）与孤立性复发组（8例）两组间中位生存时间分别为32.0个月和27.0个月（*Log-rank*检验：χ^2^=0.434，*P*=0.510）。肿瘤≤3 cm（9例）与 > 3 cm（11例）两组间中位生存时间分别为34.0个月和27.0个月（*Log-rank*检验：χ^2^=1.993，*P*=0.158）。肿瘤复发时间≤36个月（10例）与 > 36个月（10例）两组间中位生存时间分别为21.0个月和32.0个月（*Log-rank*检验：χ^2^=0.542，*P*=0.462）。

**1:**
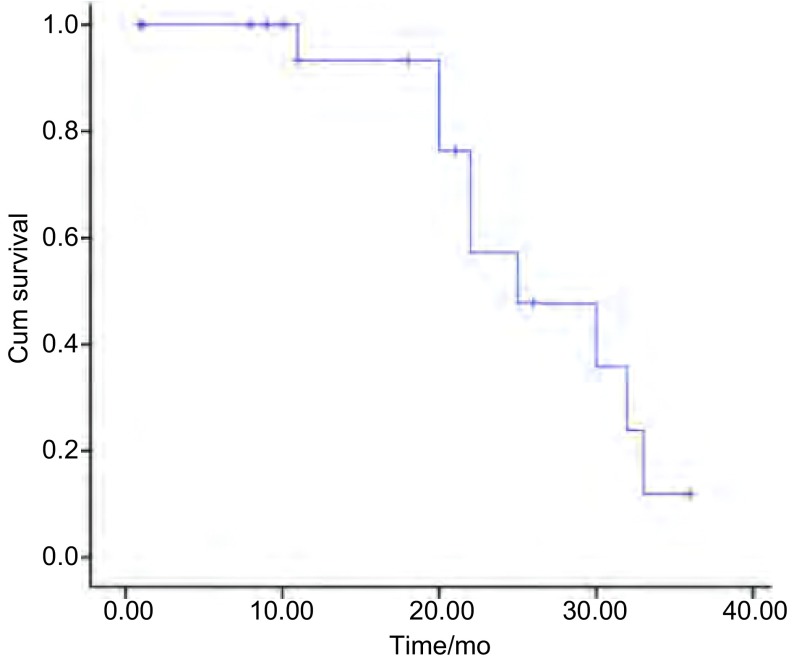
20例患者无进展生存曲线 The progression-free survival curve of all 20 patients

**2:**
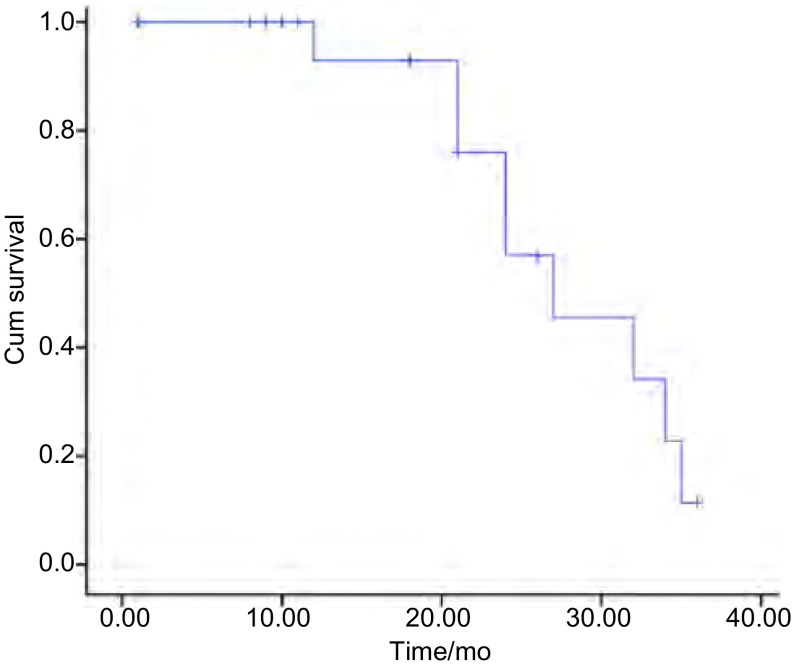
20例患者生存曲线 The cumulative survival curve of all 20 patients

## 3 讨论

长期以来，肿瘤的转移或复发即意味着晚期，然而随着人们对肿瘤转移或复发认识的不断深入，提出了寡转移（oligometastases）和寡复发（oligo-recurrence）的概念^[6]^。在第7版肺癌TNM分期中将位于同一肺叶的肿瘤划分为T3期，将位于同侧肺不同肺叶的肿瘤由M1期改为T4期^[7]^。NSCLC术后IPSLROM是否也具有类似的临床特点需要进一步研究：本组病例肺癌切除术后的复发转移时间平均63.3个月，其中转移时间平均63.0个月，复发时间平均63.6个月，无统计学差异（*P*=0.164），因此IPSLROM的特点是常发生于NSCLC术后5年以后；其次，由于术后转移复发时间长，因此诊断时年龄比较高，本组病例中12例大于70岁，平均69.2岁；再次，由于之前存在着肺切除术的问题，因此存在再次手术难度增加和低肺功能的问题，因此需要考虑治疗手段的选择。

NSCLC术后IPSLROM治疗应该在患者身体条件允许的条件下首选局部治疗^[8]^，同时辅以全身治疗以提高局部控制率。NSCLC切除术后局部复发如果局限于肺内，经过外科治疗可以获得较长的生存期^[9]^，术后5年生存率达38.3%-40.0%，手术切除率及根治率分别为75%及80%^[10]^。手术适应证包括术前评估肿物能够手术切除，术前除外纵隔淋巴结和远处转移，术前评价患者的心肺肝肾功能可耐受手术。尽管如此，从安全性和有效性来说，再手术切除存在一定的难度、并发症多，应当慎重选择。而放疗又存在放射性肺炎、支气管梗阻、气管支气管坏死、食管溃疡、肺动脉出血等并发症，对本来肺功能低下的患者来说无疑是雪上加霜，因此其适应证选择主要在靠近纵隔或肺门的复发，如支气管残端、纵隔淋巴结复发等，这些部位的放疗几乎不受呼吸运动影响，同时放疗对肺功能影响也较小^[11, 12]^。

由于NSCLC术后IPSLROM的患者存在肺功能低下和病期较晚等原因，仅有低于30%的患者适合再手术，而70%以上的患者只接受了放化疗，生存率低于再手术组，因此寻找新的局部补救治疗手段。RFA已被用于不能手术肺部肿瘤的治疗，国内外的研究^[13-15]^证明了其安全性、可行性和有效性，它具有微创、适形、并发症少、可以重复进行和效果可靠等优点，已经成为肺部肿瘤微创物理靶向治疗的首选治疗手段^[16]^。RFA是以肿瘤为靶区根除肿瘤组织及周围0.5 cm-1 cm的正常组织，最大限度地保护了正常肺组织，因此它的最佳适应证是位于肺实质内的周围型肿瘤，无疑成为NSCLC术后IPSLROM重要的治疗手段之一。

Schoellnast等^[17]^对NSCLC术后复发进行的55次消融中，平均随访28.6±20.3个月（1个月-98个月），1年、3年、5年生存率分别为97.7%、72.9%和55.7%；1年、3年无复发生存分别为76.7%和41.1%。提出射频消融可以作为复发性NSCLC的局部控制的补救手段，尤其是对 < 3 cm的肿瘤（虽然没有统计学差异）。本组病例平均随访时间19个月（1个月-36个月），中位生存时间27.0个月，1年生存率92.9%，2年生存率57%。经过统计学分析，肺内转移组与复发组，肿瘤≤3 cm与 > 3 cm，肿瘤复发时间≤36个月与 > 36个月的中位生存时间均无统计学差异，可能与病例数少有关。

本组病例均在局麻CT引导下行RFA，术中患者常会感觉局部发热、出汗、甚至心率加快，无需特殊处理发热。本组术中并发症主要是胸痛，5例发生于肺切除侧，3例发生于肺切除对侧，可能的原因是由于贴近壁层胸膜的病灶在射频消融时热传导刺激胸膜神经所致，Okuma等^[18]^单变量和多变量分析研究认为，疼痛的发生与病变距离胸壁在1 cm以内明显相关。胸痛表现为从可以忍受的轻微疼痛到重度疼痛。针对重度疼痛需要高剂量的麻醉剂或镇静剂，甚至全身麻醉；如果无效，需要降低靶温度到70 ℃，几分钟后，再逐渐升高靶温度；如果患者仍不能耐受，观察CT三维重建图像，如果有射频针接近胸膜，可以旋转射频针，再消融；如果还有疼痛，可以向胸腔内推射频针，使脏层胸膜离开壁层胸膜，即造成人工气胸^[19, 20]^。术后消融综合征常表现为发热，一般为吸收热，热程在3 d-5 d，体温在38.5 ℃以下。肿瘤较大者，发热较高，但一般不超过39 ℃。术后是否预防性抗生素治疗目前还存在争议，本组病例多为老年人、且肺功能低下，推荐预防性抗生素治疗。术后胸痛发生率约30%，以术后48 h为重，经止痛处理好转。

总之，射频消融具有微创、安全、有效等优点，适用于不能耐受手术的NSCLC术后IPSLROM局部补救治疗手段；但是由于病例数少，需要进一步研究。
